# Self-Assembly and Gelation Behavior of Methacrylated PEO–PPO–PEO Triblock Copolymer Pluronic F127

**DOI:** 10.1021/acs.langmuir.5c06879

**Published:** 2026-03-05

**Authors:** Mateus P. Bomediano, Laura C. E. da Silva, Tomás S. Plivelic, Marcelo G. de Oliveira

**Affiliations:** † Institute of Chemistry, University of Campinas, UNICAMP, Campinas, SP 13083-970, Brazil; ‡ Brazilian Nanotechnology National Laboratory (LNNano), Brazilian Center for Research in Energy and Materials (CNPEM), Campinas, SP 13083-100, Brazil; § MAX IV Laboratory, Lund University, P.O. Box 118, 221 00 Lund, Sweden

## Abstract

Methacrylated Pluronic triblock copolymers are widely used as photo-cross-linkable hydrogels, yet the effect of terminal methacrylation on self-assembly and thermoreversible gelation prior to cross-linking remains poorly understood. Here, we systematically investigate how the degree of methacrylation influences micellization, micelle packing, and gelation in Pluronic F127 using differential scanning calorimetry, rheology, synchrotron small-angle X-ray scattering (SAXS), in situ heating SAXS, and cryogenic transmission electron microscopy. Micellization thermodynamics are largely unaffected by methacrylation, with similar micellization enthalpies, temperatures, micelle core sizes, and aggregation numbers across all samples, confirming that micellization remains governed by PPO dehydration. In contrast, gelation is impacted. Increasing methacrylation shifts the gelation temperature to higher values, broadens the soft gel regime, and delays the emergence of long-range micellar order. SAXS and cryo-TEM reveal weakened intermicellar interactions, reduced corona interpenetration, and the formation of smaller, less ordered micellar domains with increasing methacrylation. Time-resolved SAXS further shows that methacrylation slows gelation kinetics under nonequilibrium heating conditions. Overall, terminal methacrylation primarily alters gelation through kinetic and structural effects rather than changes in micellization energetics. The resulting gel diagrams provide practical guidelines for designing methacrylated Pluronic hydrogels in applications requiring controlled gelation, including photo-cross-linking, additive manufacturing, drug delivery, and tissue engineering.

## Graphical abstract

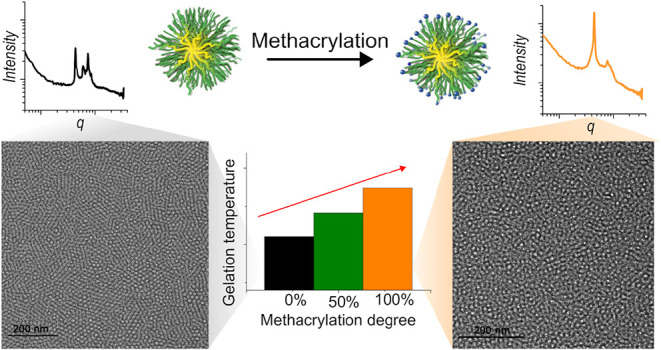

## Introduction

Water-soluble nonionic triblock copolymers of poly­(ethylene oxide)-poly­(propylene oxide)-poly­(ethylene oxide) (PEO–PPO–PEO), commercially known as Pluronic, Poloxamer, or Synperonic, are available in a range of different PEO and PPO block sizes.^1,2^ The terminal PEO blocks of Pluronic are hydrophilic, while their central PPO blocks are more hydrophobic, conferring to the molecule as a whole an amphiphilic behavior. Pluronic solutions spontaneously form micelles above the critical micelle concentration (*cmc*) and critical micelle temperature (*cmt*). The micellization process is endothermic and entropy-driven, primarily resulting from PPO block dehydration.^3–6^ At higher temperatures and concentrations, partial dehydration of PEO blocks leads to water segregation into the intermicellar space, promoting micelle packing and ultimately gelation.^7,8^ The thermoreversible micellization and gelation behavior of Pluronics, along with their biocompatibility have allowed their application in drug delivery,^9–13^ biomaterials,^14–17^ and 3D printing.^18–23^

Pluronic F127 (PEO_100_–PPO_65_–PEO_100_) (F127) is the most used Pluronic in pharmaceutical applications due to its biocompatibility and sol–gel transition under body temperature.^2,24^ Recently, the interest in the use of hydrogels that can be fabricated into constructs of specific geometries for tissue engineering applications has led to the development of strategies for obtaining mechanically stable Pluronic hydrogels.^22,25^ One of such strategies is the functionalization of the terminal hydroxyl groups of the PEO blocks with vinyl groups to allow their further photochemical cross-linking. Several reports have described the modification of F127 with methacrylate terminal groups (dimethacrylated Pluronic F127, F127-DM) for obtaining 3D printable hydrogels^20,21,26–29^ or to promote in situ gelation.^30,31^ So far, these studies have focused exclusively on F127-DM and its combination with other acrylated polymers, such as poly­(acrylic acid) and methacrylated hyaluronic acid. While these studies have explored the use of photo-cross-linked Pluronic-DM hydrogels, the influence of methacrylate end groups on micellization and gelation properties, and nanostructural organization remains unclear in the literature. From a conceptual standpoint, replacing the terminal hydroxyl groups of Pluronic PEO blocks with methacrylate groups eliminates the hydrogen bonds that these hydroxyl groups form with water in aqueous solutions of native Pluronic. Since the hydroxyls of the PEO blocks are located on the outside of the micelle crown, interacting directly with the water in the intermicellar spaces, it is expected that methacrylation will affect, to some extent, both the geometry and the dynamics of micelle packing in the gelation process. In addition, methacrylate groups are significantly more voluminous, and therefore, the gelation process may also be affected by steric factors. In fact, in a previous study, using small-angle X-ray scattering (SAXS) measurements, we demonstrated that methacrylation significantly affects the recovery of the Pluronic micellar arrangement after extrusion.^20^

Herein, we report a detailed study of the micellization and gelation processes of F127-DM, using a combination of rheology, differential scanning calorimetry (DSC), cryogenic transmission electron microscopy (cryo-TEM), and synchrotron small-angle X-ray scattering (SAXS) techniques. The results obtained allowed us to characterize the effect of methacrylation on the micellar nanostructure of F127 with methacrylation degree (MD) 50 and 100%. Our findings demonstrate that, while micellization follows a behavior similar to native Pluronic, an increase in the MD introduces disruptions in the gelation process, leading to smaller clusters of ordered micelles, increasing gelation temperature. Integration of the obtained results allowed the first description of the gel diagram of F127-DM. From the application standpoint, the understanding of the phase behavior of F127-DM underscores the development of new applications, based on 3D printing of hydrogel constructs for drug delivery and tissue engineering, where the tunability of gelation may be critical.

## Experimental Section

### Materials

Pluronic F127 - poly­(ethylene oxide)_100_-poly­(propylene oxide)_65_-poly­(ethylene oxide)_100_ (Molar Mass: 12 600 Da) (F127), methacrylic anhydride, methacrylic acid, tetrahydrofuran (THF), petroleum ether, deuterated dimethyl sulfoxide (*d*_6_-DMSO) were purchased from Sigma-Aldrich Chem. Co. and used without further purification. Solutions were prepared with ultrapure Milli-Q water (resistivity 18.2 MΩ.cm).

#### Pluronic Methacrylation

Pluronic F127 was methacrylated by direct reaction with methacrylic anhydride in the absence of solvent, based on the procedure reported by Bomediano et al.^22^ In short, appropriate amounts of F127 and methacrylic anhydride were subjected to 10 heating cycles of 30 s duration per cycle, with 30 s periods of manual rotary shaking between each cycle. The methacrylation reaction was carried out at 65 °C, ensuring the polymer remained in the molten state throughout the reaction. The products were dissolved in THF, precipitated in petroleum ether, vacuum filtered in a Buchner funnel, and dried under N_2_ flow for 3 h. After drying, the products were kept in the refrigerator, protected from light. Two different MD were obtained using the molar ratios of methacrylic anhydride: terminal hydroxyl groups of F127, 1:1 (MD 50%, F127-DM50) and 5:1 (MD 100%, F127-DM100).

#### Proton Nuclear Magnetic Resonance (^1^H NMR) Spectrometry

^1^H NMR spectra were obtained in a Bruker AVANCE 400 NMR spectrometer operating at 400 MHz, using DMSO-*d*_6_. 64 scans were collected. The methacrylation degree (MD), defined by the percentage of terminal hydroxyl groups of the PEO blocks that were esterified by methacrylic anhydride, generating methacrylated end groups, was estimated based on previous studies.^22^

#### Vibrational Spectroscopy

Samples were characterized by Attenuated Total Reflectance Fourier Transformed Infrared Spectroscopy (ATR-FTIR) using an Agilent-CARY 630 spectrophotometer in the wavenumber range of 400 to 4000 cm^–1^ with 1 cm^–1^ resolution and 64 scans per spectrum. Dry powder samples of native F127 and F127-DM were placed directly onto the ATR crystal and gently pressed to ensure good contact prior to data collection.

#### Preparation of F127 and F127-DM Solutions

Aqueous F127 and F127-DM solutions were prepared by the cold method^32^ by slowly adding the dry polymers in powder form, over Milli-Q water under magnetic stirring in an ice bath. The polymeric solutions obtained were kept under refrigeration, protected from light.

#### Differential Scanning Calorimetry

The thermal properties of aqueous F127 and F127-DM solutions were analyzed by differential scanning calorimetry (DSC), using a DSC-Q100 calorimeter (TA Instruments). Initially, a heating rate of 5 °C min^–1^ from 5 to 50 °C was used. Subsequently the samples were cooled to 5 °C at a cooling rate of 5 °C min^–1^ and subjected to a second heating under identical conditions. The micellization enthalpy (Δ*H*_m_) of the solutions was calculated through the integration of the endothermic peak of the second heating thermograms, the associated errors were determined from the standard deviation of Δ*H*_m_ values normalized by the molar quantities of F127. The micellization temperature (*T*_mic_) was extracted from the intercept of the tangent lines to the two flanks of the endothermic peaks.

#### Rheology

Rheological properties of aqueous solutions of F127 and F127-DM were evaluated using a HAAKE Mars 40 rheometer with parallel plate geometry (1 mm gap). Amplitude sweep measurements were first performed to determine the linear viscoelastic region (LVER) (Figure S1, Supporting Information). Subsequently, oscillatory temperature sweep experiments were carried out at a fixed frequency of 1 Hz and a constant stress of 1 Pa, while the temperature was increased at a linear heating rate of 1 °C min^–1^. A solvent trap was used to avoid water evaporation. The sol–gel transition temperature (*T*_gel_) was defined as the crossover point between the storage modulus (*G*′) and the loss modulus (*G*″).

#### Cryogenic Transmission Electron Microscopy

Cryo-TEM was performed under low-dose conditions using a TALOS F200C transmission electron microscope (Thermo Fisher Scientific) operating at 200 kV and equipped with a 4k × 4k CETA D CCD camera. Samples were vitrified in liquid ethane according to the procedure described by da Silva et al.^33^

#### Small Angle X-ray Scattering

Micellar organization was characterized as a function of temperature using small-angle X-ray scattering (SAXS). Measurements were performed at the CoSAXS beamline, MAXIV Laboratory.^34^ Samples were loaded into 1.5 mm diameter quartz capillaries mounted in a temperature-controlled holder connected to a thermal bath. Data were collected by vertically scanning the capillaries, with an exposure time of 0.2 s per position; the average of 20 positions was used for analysis. Temperature was varied from 10 to 50 °C in 5 °C increments. A wavelength of λ = 1.0 Å and a sample-to-detector distance of 3.456 m were used. Scattering patterns were recorded with an Eiger2 4M detector (Dectris), yielding a *q*-range of 0.0034–0.26 Å^–1^, where *q* = 4π/λ sin­(θ) and 2θ is the scattering angle.

Complementary SAXS measurements were conducted at the CATERETÊ beamline, Sirius Synchrotron Laboratory (LNLS/CNPEM). Samples were mounted in a capillary holder equipped with a Peltier heating system. A wavelength of 1.378 Å and a sample-to-detector distance of 1.7 m were used, providing a q-range of 0.00282–0.34 Å^–1.^ Two-dimensional SAXS images were recorded using a PiMEGA detector (3072 × 3072 array, PITEC).

#### SAXS Data Treatment

For 10 wt % polymer systems of micelles in water solution, SAXS intensity profiles were analyzed using the form factor of block copolymer micelles^35,36^ combined with a structure factor of a hard-sphere Percus–Yevick approximation.^37^ Further details are described in Supporting Information. Data fitting and simulations were performed using SasView 6.0.0.

For micelles arranged in a three-dimensional cubic lattice, the relationship between the scattering vector and the unit cell dimension was determined using eq 1:^38^

1 $$\frac{2\pi }{q^{*}}=d_{\mathrm{hkl}}=\frac{a}{\sqrt{h^{2}+k^{2}{+l}^{2}}}$$

where q* is the position of the first reflection, *a* is the unit cell parameter and *hkl* are the Miller indices.

In a body center cubic (bcc) structure, each unit cell contains two micelles. The aggregation number (*N*_agg_), was therefore calculated according to eq 2:

2 $$N_{\mathrm{agg}}=\frac{a^{3}CN_{A}}{2M_{W}}$$

where *C* is the polymer concentration (w/v), *N*_A_ is the Avogadro number, *M*_w_ is the molecular weight of polymer, and *a* ^3^/2 is the number density for a bcc lattice.

For a face centered cubic (fcc) structure, the unit cell contains four micelles, and the aggregation number is calculated as eq 3:

3 $$N_{\mathrm{agg}}=\frac{a^{3}CN_{A}}{4M_{W}}$$

Assuming a spherical PPO core, the core radius (*R*_c_) was calculated using eq 4:

4 $$R_{c}=\sqrt[{3}]{\frac{3N_{\mathrm{agg}}M_{\mathrm{PPO}}}{4\pi N_{A}\rho_{\mathrm{PPO}}}}$$

where *M*_PPO_ is the molecular weight of the PPO block and ρ_PPO_ is the density of PPO.

## Results and Discussion

### F127-DM: Synthesis and Characterization

Figure 1a shows the methacrylation of Pluronic F127 to produce Pluronic F127 dimethacrylate (F127-DM). Figure 1b presents a schematic illustration of the native and methacrylated PEO–PPO–PEO chains and their corresponding micelles and micelles packing (hydrogel formation) under heating, emphasizing the hydrophobic PPO core and the hydrophilic corona formed by either unmodified PEO or methacrylated PEO.^5,39^ To evaluate how terminal hydroxyl group modification influences the material behavior, two MD were employed: approximately 50% methacrylation (F127-DM50) and full methacrylation (F127-DM100). Representative ^1^H NMR spectra of native F127, F127-DM50, and F127-DM100 are shown in Figure 1c.

**Figure 1:**
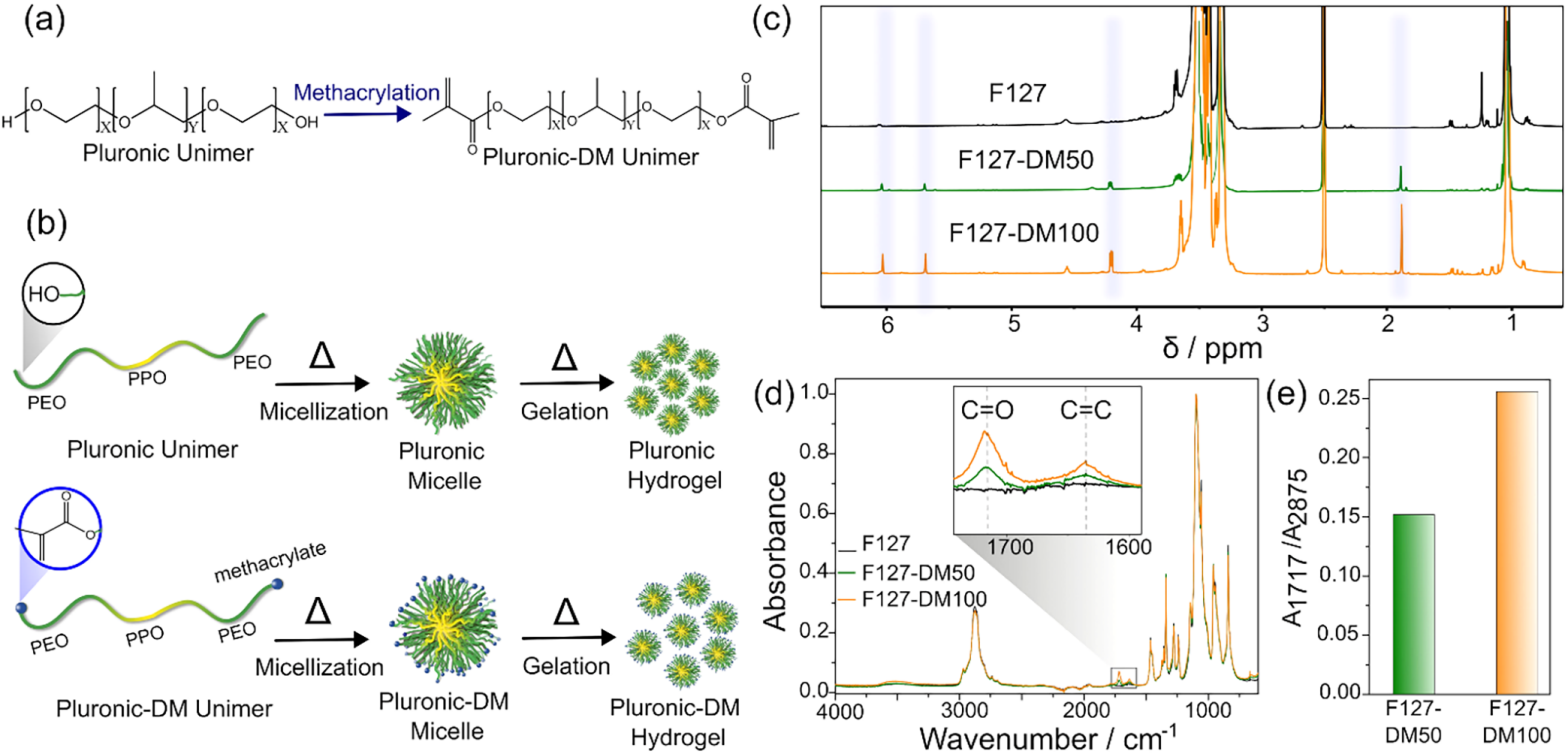
(a) Methacrylation of Pluronic to form Pluronic dimethacrylate (Pluronic-DM). (b) Schematic representation of individual polymer chains and their self-assembling into micelles, followed by micelles packing and gelation under heating. The relative numbers of PEO and PPO repeat units in F127 (*X* = 100, *Y* = 65) are not drawn to scale. (c) ^1^H NMR spectra of F127, F127-DM50, and F127-DM100; the blue-shaded region highlights protons from the methacrylate end groups. (d) FTIR spectra of F127, F127-DM50, and F127-DM100. (e) Ratio of absorbance intensities at 1717 and 2875 cm^–1^ (*A*_1717_/*A*_2875_) for F127-DM50, and F127-DM100.

In all spectra, resonances between 3.30 and 3.51 ppm arise from methylene protons in both the PPO and PEO blocks, while the peak at 1.03 ppm corresponds to methyl protons in the PPO block. Successful methacrylation is indicated by new signals at 6.03 and 5.70 ppm, assigned to vinyl protons, at 1.88 ppm from methacrylate methyl protons, and at 4.20 ppm from PEO methylene protons adjacent to the methacrylate group.^22^ The calculated methacrylation degree for F127-DM50 and F127-DM100 are summarized in Table 1.

**Table 1:** Pluronic F127-DM Samples and Methacrylation Degree (MD) Estimated by ^1^H NMR

Sample name	MD (%)
F127-DM50	54 ± 3
F127-DM100	95 ± 2

Figure 1d presents representative FTIR spectra of native F127, F127-DM50, and F127-DM100. New absorption bands at 1717 cm^–1^, assigned to carbonyl stretching, and at 1636 cm^–1^, corresponding to C = C stretching, are observed in the methacrylated samples, confirming the presence of methacrylate groups. Figure 1e compares the ratios of infrared absorbance at 1717 cm^–1^ to that at 2875 cm^–1^, the latter corresponding to C–H stretching and used as an internal reference. The higher *A*_1717_/*A*_2875_ ratio observed for F127-DM50 relative to F127-DM100 is consistent with higher MD determined by ^1^H NMR.

### Micelle Formation

Figure 2a–c show the thermograms for F127, F127-DM50, and F127-DM100 at concentrations 10, 20, and 30 wt %. All solutions exhibited an endothermic peak, associated with the progressive dehydration of the PPO blocks upon heating, which drives micellization, as commonly observed in Pluronic solutions.^8,35,40^ Figure S2a and b (Supporting Information) summarize the micellization enthalpy (Δ*H*_m_) and the temperature at the peak of micellization (*T*_mic_), respectively. The Δ*H*_m_ values were normalized by the molar amount of polymer to allow direct comparison across samples and concentrations. No statistically significant differences in Δ*H*_m_ were observed between native F127 and the methacrylated derivatives. Although *T*_mic_ decreases with increasing polymer concentration, as expected for PEO–PPO–PEO triblock copolymers, no systematic dependence on the degree of methacrylation was detected. Together, these results indicate that methacrylation does not substantially affect the thermodynamics of micellization within the concentration range studied. This outcome is expected, as micellization in Pluronics is primarily governed by dehydration of the PPO block, which is not modified during methacrylation.^5,35^

**Figure 2:**
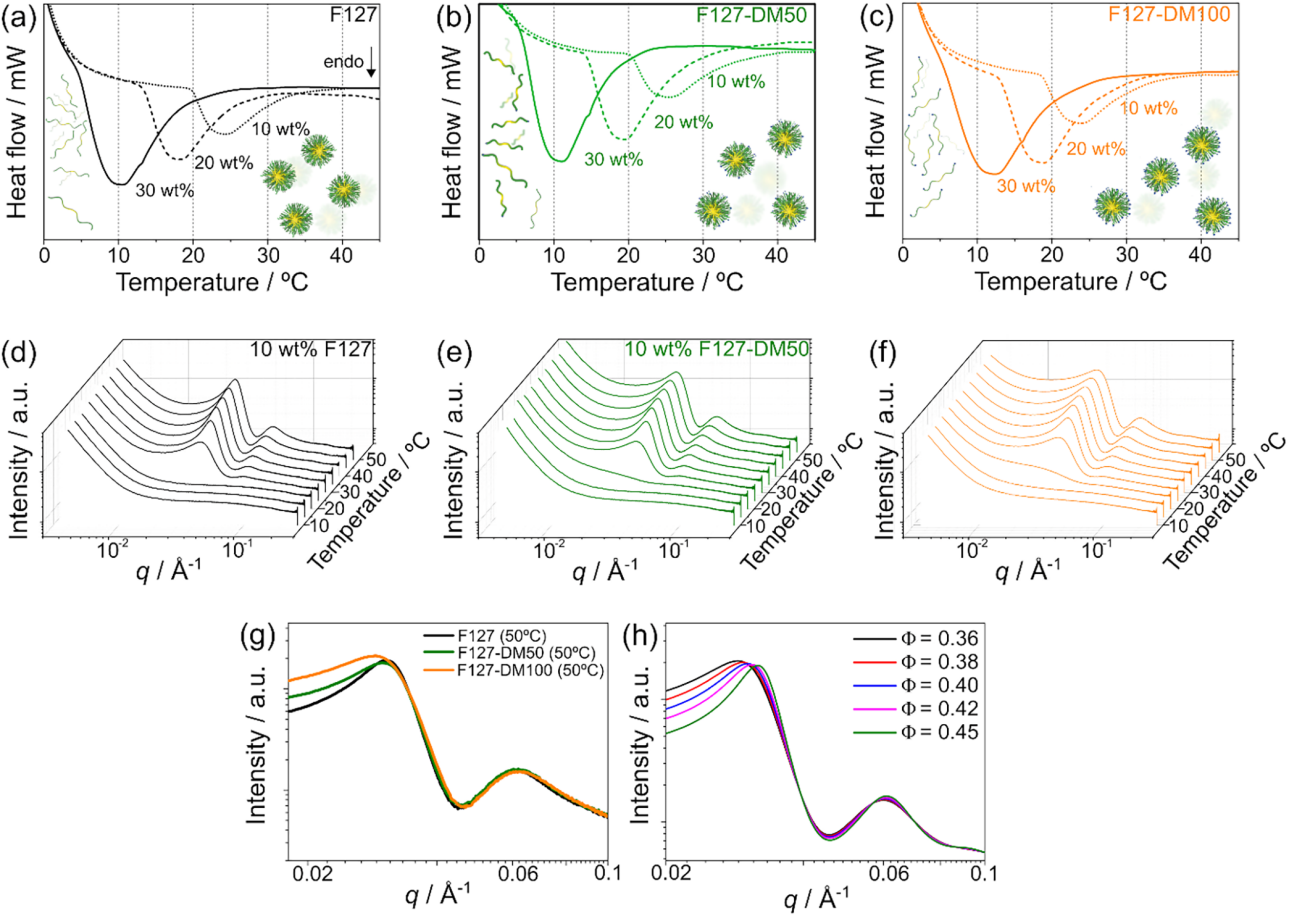
DSC thermograms of F127, F127-DM50, and F127-DM100 at polymer concentrations of (a) 10 wt %, (b) 20 wt %, and (c) 30 wt %. Three-dimensional representations of the temperature-dependent SAXS profiles for 10 wt % solutions of (d) F127, (e) F127-DM50, and (f) F127-DM100, collected from 10 to 50 °C in 5 °C increments. (g) SAXS intensity profiles of 10 wt % F127, F127-DM50, and F127-DM100 at 50 °C. (h) Simulated SAXS curves generated using a block copolymer micelle form factor combined with a hard-sphere structure factor based on the Percus–Yevick approximation, with the micellar effective volume fraction varied (Φ = 0.36–0.45; see Supporting Information).

Figure S2c–e show the full width at half-maximum (FWHM) of the micellization peaks as a function of methacrylation degree at each concentration. At 10 and 20 wt %, the FWHM remains unchanged across samples. In contrast, a clear increase in peak width is observed at 30 wt % with increasing methacrylation. This broadening suggests a kinetic effect, in which methacrylate end groups slow the self-assembly process, leading to a less abrupt transition. The broader peaks likely reflect a wider distribution of intermediate structures or transient heterogeneity during micelle formation. Because micellization kinetics depend on polymer diffusion, which is increasingly hindered at higher concentrations, this effect becomes evident only at 30 wt % F127.

To evaluate how methacrylation affects the micellar structure of F127, SAXS measurements were collected for 10 wt % solutions of F127, F127-DM50, and F127-DM100 over a temperature range from 10 to 50 °C (Figure 2d–f). At 10 and 15 °C, the scattering curves for all samples show a smooth decay at high *q*, characteristic of dispersed polymer chains in the sol state.^41^ At 20 °C, native F127 shows little change compared to lower temperatures, whereas F127-DM100 displays a broad scattering feature, indicating the onset of micelle formation. F127-DM50 exhibits intermediate behavior between these two cases.

At 25 °C, a pronounced peak at *q* ≈ 0.062 Å^–1^ appears in all samples and is attributed to the micellar form factor. Increased scattering intensity at low *q* suggests the emergence of intermicellar correlations.^36,42^ This temperature range corresponds closely to the micellization transition identified by DSC (Figure 2a–c). As the temperature increases from 25 to 50 °C, the overall scattering intensity rises, while the low-*q* intensity decreases slightly, consistent with the formation of a large population of interacting micelles.

Figure 2g compares the SAXS profiles of all samples at 50 °C. An increase in low-*q* intensity with higher MD suggests changes in intermicellar interactions. To interpret this trend, simulated SAXS curves were generated using a block copolymer micelle form factor combined with a hard-sphere structure factor^36,42^ (Figure 2h). In these simulations, all structural parameters were held constant while the effective volume fraction (Φ) was varied. Φ is interpreted as an effective volume fraction, reflecting intermicellar interactions such as steric repulsion, micellar softness, and corona chain overlap, rather than the actual physical volume fraction of the micelles. The results show that increasing Φ leads to a reduction in low-*q* intensity. Comparison with the experimental data therefore indicates that higher methacrylation weakens intermicellar interactions, effectively lowering the effective volume fraction. This behavior is attributed to the replacement of terminal hydroxyl groups with methacrylate moieties, which reduces hydrogen bonding between PEO chain ends and water within the intermicellar regions. As a result, intermicellar connectivity is diminished. Fitting of the SAXS data supports this interpretation (Supporting Information, Figure S3): the micelle core radius (*R*_c_) and aggregation number (*N*_agg_) remain nearly constant across samples, whereas Φ decreases systematically with increasing degree of methacrylation, as summarized in Table 2.

**Table 2:** Micellar Core Radius (*R*_c_), Aggregation Number (*N*_agg_), and Effective Volume Fraction (Φ) Obtained from SAXS Fits for 10 wt % Solutions of F127, F127-DM50, and F127-DM100 at 50 °C

Sample	*R*_*c*_	*N*_agg_	Φ
F127	4.1	74	0.41
F127-DM50	4.2	74	0.38
F127-DM100	4.0	75	0.31

Although methacrylate groups are less hydrophilic than hydroxyl groups and could, in principle, be expected to partition into the micelle core, the unchanged core radius indicates that they preferentially remain in the corona.

### Gelation Kinetics

To examine the effect of methacrylation on gelation, DSC thermograms of 30 wt % solutions of F127, F127-DM50, and F127-DM100 are shown in Figure 3a, with an inset highlighting the 5–20 °C range. In addition to the broad endothermic peak associated with micellization, a second weaker endothermic transition is observed in all samples and is attributed to gelation.^8,41^ Arrows mark the endothermic event corresponding to the critical gelation temperature (*cgt*). As MD increases, the *cgt* shifts to higher temperatures, indicating that methacrylation delays gel formation, likely by reducing intermicellar interactions.

**Figure 3:**
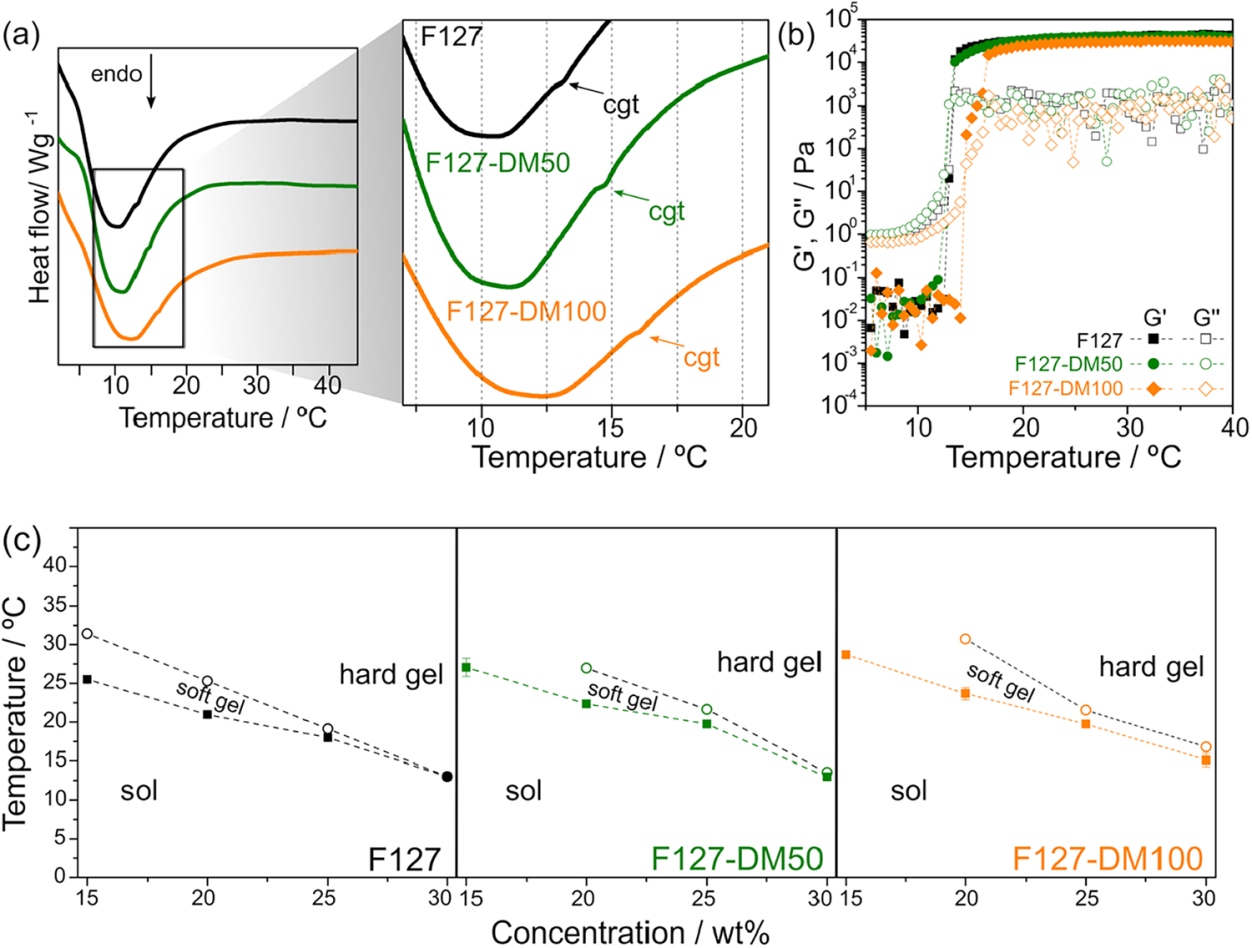
(a) DSC thermograms of 30 wt % F127, F127-DM50, and F127-DM100, with the inset highlighting the critical gelation temperature (*cgt*). (b) Storage modulus (*G′*) and loss modulus (*G*″) as a function of temperature for 30 wt % F127, F127-DM50, and F127-DM100. (c) Gel diagram for aqueous solutions of native F127, F127-DM50, and F127-DM100. Data points were obtained from temperature sweep measurements performed in a stress-controlled rheometer at a constant stress of 1 Pa, from 5 to 40 °C (see Figure S4, Supporting Information). Filled symbols (■) indicate the temperature at which *G′* first exceeds *G″*, while open symbols (○) correspond to the temperature at which *G′* reaches its plateau value at each concentration.

This trend was further examined using rheology. Gelation in Pluronic systems corresponds to a disorder-to-order transition, characterized by a sharp increase in viscosity as the solution transforms into a nonflowing hydrogel.^5^ With increasing temperature, enhanced micellar interactions promote ordering into mesophases.^43^ The sol–gel transition temperature (*T*_gel_) was determined rheologically from the crossover point where the storage and loss moduli are equal (*G′* = *G″*).^7^ Figure 3b shows *G′* and *G″* as a function of temperature for 30 wt % F127, F127-DM50, and F127-DM100, confirming that *T*_gel_ increases with increasing methacrylation. Temperature sweeps of F127, F127-DM50, and F127-DM100 at concentrations of 10, 15, 20, 25, and 30 wt % are shown in Figure S4, Supporting Information. The cgt values obtained from DSC closely match the rheologically determined *T*_gel_ values, as summarized in Table S1 (Supporting Information). While both temperatures describe the sol–gel transition, DSC thermograms identify the thermal event associated with gelation, which occurs over a temperature interval, whereas rheological measurements probe the evolution of mechanical properties accompanying the sol–gel transition. Minor differences between the two techniques are attributed to differences in heating rates, which can affect gelation kinetics but do not alter the observed effect of methacrylation.

The gel diagrams of F127, F127-DM50, and F127-DM100 are summarized in Figure 3c. Because gelation is more clearly identified by changes in viscoelastic properties than by the weak endothermic signal observed in the DSC thermograms, the boundary lines separating the different regions in Figure 3c were defined based on the gelation temperatures (*T*_gel_) extracted from rheological measurements (Figure S4, Supporting Information).

Three distinct regions are identified in the gel diagrams (Figure 3c): (i) a sol region, where the loss modulus exceeds the storage modulus (*G″* > *G′*); (ii) a soft gel region, which begins at the crossover point (*G′* = *G″*) and extends until *G′* and *G″* reach plateau values (*G′* > *G″*); and (iii) a hard gel region, established once these plateau moduli are attained.^44,45^ These regions are illustrated in Figure S5, Supporting Information, for native F127.

At 15 wt %, the lowest concentration at which gelation is observed, F127-DM50 and F127-DM100 form only a soft gel, with no hard-gel plateau detected. In contrast, native F127 exhibits a well-defined hard-gel plateau, with *G′* at 40 °C approximately five times higher than that of the methacrylated samples. This result indicates that methacrylation hinders the development of long-range micellar ordering.

A control experiment was performed on a 15 wt % F127 solution heated from 5 to 40 °C at 1 °C min^–1^. After reaching the *G′* = *G″* crossover temperature, the system was held isothermally for 20 min. As shown in Figure S6 Supporting Information, both *G′* and *G″* continued to increase under isothermal conditions and resumed a faster rise once heating at 1 °C min^–1^ was restarted, until plateau values were reached. This behavior demonstrates that a heating rate of 1 °C min^–1^ does not allow the system to evolve under equilibrium conditions. Consequently, decreasing the heating rate is expected to reduce the width of the soft gel region in the phase diagrams (Figure 3c). In the limit of quasi-equilibrium heating, this intermediate region should vanish, and only a direct transition from the sol to the hard gel phase would be observed.

Although the extent of the soft gel region is heating-rate dependent, we consistently observed that the temperature interval between the *G′* = *G″* crossover and the establishment of the final plateau increase with increasing MD. The systematic broadening of this interval with increasing MD therefore reflects an intrinsic modification of gelation kinetics induced by the methacrylate groups.

The broadening of the FWHM of the endothermic micellization peak in the DSC thermograms reflects changes in micelle formation. Gelation is a cooperative process that occurs when the micelle number density and effective occupied volume permit sufficient intermicellar contacts to form a percolated network.^46^ Therefore, the substitution of the terminal hydroxyl groups by methacrylate groups affects not only micelle formation but also the cooperative long-range micellar ordering that governs gelation. In this context, the rheological shift of *T*_gel_ to higher temperatures corroborates the FWHM broadening observed in DSC, as both techniques indicate reduced cooperativity and slower self-assembly kinetics in F127-DM.

For native F127, SAXS studies have shown that the soft gel phase consists of mixed structures, including isotropic micellar clusters and small crystalline domains, whereas the hard gel phase exhibits long-range micellar order with well-defined Bragg peaks.^45,47^ The broader range of soft gel region and the increase in T_gel_ observed with increasing methacrylation indicate that terminal modification hinders the development of long-range micellar order. A similar effect was reported by Picheth et al.,^41^ who observed reduced short-range micellar ordering in mercaptopropionate-terminated F127. Together, these findings demonstrate that chemical modification of the terminal PEO blocks significantly influences the gelation process and structural organization of Pluronic F127.

### Gel Morphology

To examine gelation at the nanoscale, Figure 4a shows SAXS profile of 30 wt % of F127, F127-DM50, and F127-DM100 at 15, 25, and 50 °C. Complementary temperature-resolved SAXS data for 20 and 30 wt % samples over the range of 10 to 50 °C are provided in Figure S7 (Supporting Information). At 15 °C, distinct Bragg peaks are observed for F127 and F127-DM50, indicating the onset of micellar ordering, whereas no clear Bragg peaks are detected for F127-DM100, suggesting that long-range order is not yet established in the fully methacrylated system. This lack of long-range order in F127-DM100 is consistent with weakened intermicellar interactions arising from the methacrylate moieties.

**Figure 4:**
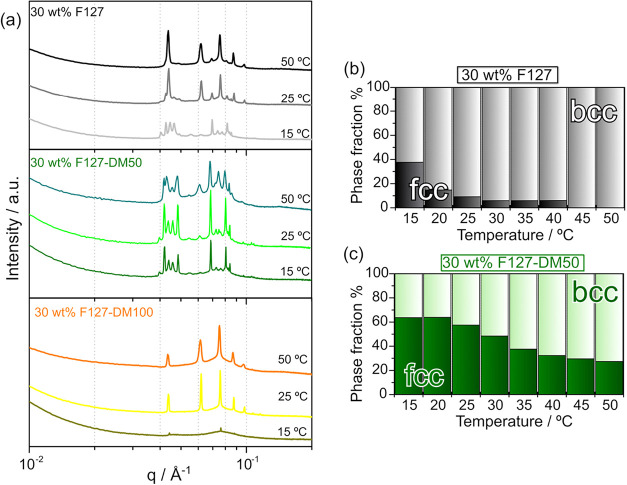
(a) SAXS profiles of 30 wt % solutions of F127, F127-DM50, and F127-DM100 at 15, 25, and 50 °C. Temperature-dependent phase fractions of body-centered cubic (bcc) and face-centered cubic (fcc) micellar packings for 30 wt % (b) F127 and (c) F127-DM50.

Regarding F127 and F127-DM50, the positions of the Bragg peaks follow the ratio $\frac{q_{i}}{q^{*}}$ = 1:√2:√3:√4, which is characteristic of a body-centered cubic (bcc, *Im*3*m*) lattice. Additional peaks are also observed at *q*-value ratios of $\frac{1}{\surd 3}$ : $\frac{\surd 4}{\surd 3}$ : $\frac{\surd 8}{\surd 3}$ : $\frac{\surd 11}{\surd 3}$ , indicating the coexistence of a face-centered cubic (fcc, *Pn*3*m*) micellar arrangement.^43^ These results show that both bcc and fcc phases are present across all samples. Notably, the appearance of Bragg peaks coincides with the sol–gel transition identified by rheology, confirming that the formation of long-range micellar order corresponds to the hard gel phase and occurs at similar temperatures. The unit cell parameters (a) for both bcc and fcc phases in 20 and 30 wt % F127-based systems at 25 and 50 °C were extracted from the SAXS data and are summarized in Table S2 (Supporting Information). For the bcc phase, *a* ≈ 22.0 nm at 20 wt % and *a* ≈ 20.3 nm at 30 wt %, while the fcc phase exhibits *a* ≈ 27.8 nm at 20 wt % and *a* ≈ 25.8 nm at 30 wt %. No significant differences were observed between native and methacrylated samples. Similarly, the aggregation number (*N*_agg_) showed no systematic dependence on the MD.

At 25 °C, the ordered fraction of native F127 is dominated by the bcc phase, whereas F127-DM50 contains a larger fraction of the fcc phase. In contrast, F127- is primarily bcc at this temperature. Upon heating to 50 °C, the intensity of the bcc-related peaks increases, while the fcc peaks diminish, indicating a temperature-driven transition toward bcc ordering. Figure 4b shows the phase fractions for F127, and Figure 4c shows those for F127-DM50, confirming that both systems undergo a transition from fcc to bcc dominance as temperature increases. The relative amounts of each phase within the ordered fraction were estimated from the areas of the first Bragg peaks associated with the bcc and fcc structures (*A*_bcc_ and *A*_fcc_, respectively), according to eq 5:

5 $$\mathrm{Fraction}\;\mathrm{bcc}\;\mathrm{phase}=\frac{A_{\mathrm{bcc}}}{A_{\mathrm{bcc}}+A_{\mathrm{fcc}}}$$

Native F127 contains two terminal hydroxyl groups, and partial methacrylation therefore produces a distribution of polymer species rather than a single, uniform structure. By accounting for the presence of diblock impurities and applying a probability-based model, we estimated the composition of F127-DM50.^48^ This sample consists of chains with zero, one, or two hydroxyl groups converted to methacrylate in approximate proportions of 21, 50, and 29%, respectively. The corresponding contributions from diblock chains are 2.1, 4.8, and 2.8% (Supporting Information, Figure S8a). As a result, a nominal methacrylation degree of 50% yields a heterogeneous population of chains with varying end-group modifications, increasing structural disorder and explaining the absence of a clear, systematic phase behavior.

In contrast, F127-DM100 is considerably more uniform. Approximately 81.5% of the chains have both hydroxyl groups methacrylated, 8.6% contain only one modified end group, and only 0.2% remain unmodified. The corresponding diblock fractions are 8.75, 0.92, and 0.02%, respectively. Detailed calculations and the complete distribution of species are provided in Supporting Information, Figure S8b and c. The coexistence of bcc and fcc phases in Pluronic-DM gels is not unusual and has been reported previously using SAXS and photo-cross-linking approaches. In these studies, both bcc and fcc structures were observed even at 30 wt % F127-DM100. At early stages of gelation and in the absence of shear, however, only the bcc phase was detected for both F127-DM50 and F127-DM100.^20^ Mortensen et al.^43^ discussed fcc–bcc transitions in terms of intermicellar interactions and diblock content. In the present system, however, the diblock fraction remains relatively small (≈9.7%), and no systematic correlation between the MD and the dominant crystalline phase is observed.

To examine how methacrylation affects gelation kinetics, time-resolved SAXS measurements were carried out during in situ heating at a rate of 5 °C min^–1^ for 20 wt % (Figure 5a and b) and 30 wt % solutions of F127 and F127-DM100 (Figure S9, Supporting Information). Unlike the equilibrium SAXS measurements discussed above, these experiments capture the dynamic evolution of structure during gelation. In both concentrations, methacrylation shifts the onset of micellar ordering to higher temperatures, as indicated by the appearance of the first Bragg peaks. At 20 wt %, Bragg peaks emerge at approximately 23 °C for F127 and 26 °C for F127-DM100. At 30 wt %, the corresponding onset temperatures are about 14 and 17 °C, respectively. These results show that methacrylation delays the development of long-range micellar order during heating, consistent with the higher gelation temperatures and slower ordering kinetics observed in DSC and rheological measurements.

**Figure 5:**
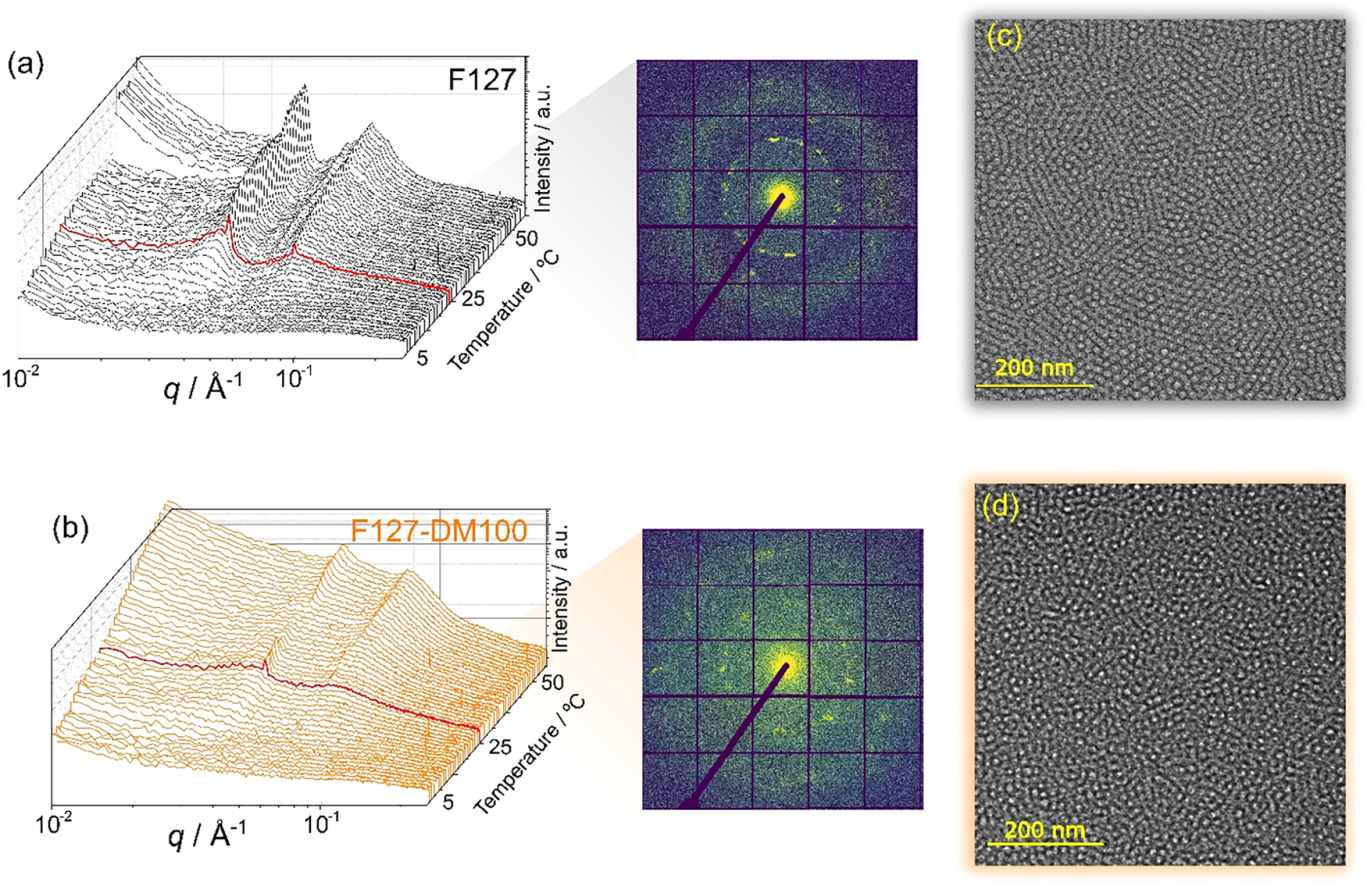
Three-dimensional representation of the temperature-dependent evolution of SAXS profiles during in situ heating of 20 wt % (a) F127 and (b) F127-DM100. 2D SAXS pattern associated with the last curve of the heating of 20 wt % F127 and F127-DM100 are shown on the right. Cryo-TEM micrographs of 20 wt % solutions of (c) F127 and (d) F127-DM100.

These results confirm that methacrylation increases the gelation temperature even under nonequilibrium conditions. Beyond the temperature shift, the heating kinetics provide further insight into the ordering process. The broader Bragg peaks observed for F127-DM100 compared to F127 indicate reduced long-range order, likely reflecting smaller clusters of ordered micelles. This interpretation is consistent with the higher low-*q* scattering intensity, which suggests increased structural disorder and weaker intermicellar correlations in the methacrylated system.^20^

For 30 wt %, a transition from fcc to bcc is observed with increasing temperature, whereas for F127-DM100, exclusively bcc Bragg peaks are detected, consistent with SAXS data under equilibrium conditions. For 20 wt % F127, both bcc and fcc Bragg peaks are clearly visible, with bcc reflections being more intense, particularly at higher temperatures. In contrast, peak identification for F127-DM100 is more challenging due to weaker signals, broader peaks, and increased noise.

Analysis of the two-dimensional SAXS patterns at 53 °C further highlights these differences. For F127, well-defined spots appear along the diffraction rings, particularly in the innermost ring, indicating ordered micellar domains with random orientations. By comparison, the SAXS pattern of F127-DM100 displays fewer and more diffuse spots, consistent with less uniform micellar spacing and the presence of smaller, less ordered clusters.

Figure 5c and d show cryo-TEM micrographs of 20 wt % solutions of F127 and F127-DM100. Cryo-TEM micrographs at lower magnification for 20 wt % F127 and F127-DM100 are shown in Figure S10, Supporting Information. In both cases, the presence of parallel rows reflects the superposition of ordered micelles.^33,41^ However, clusters containing these ordered rows are markedly larger for native F127 than for F127-DM100, as shown in Figure S11 (Supporting Information) for 30 wt % solutions, and is in good agreement with the SAXS observations. Direct visualization of these micellar arrangements therefore supports the conclusion that increasing methacrylation disrupts micelle packing by increasing the number of clusters and decreasing their size, which ultimately means a decrease in the length of the long-range ordering.

According to Dormidontova, water molecules confined between PEO chains can act as physical cross-linking agents, thereby promoting gelation.^49^ In native F127, PEO–PEO physical cross-linking may occur either through direct hydrogen bonding between terminal hydroxyl groups or via water-mediated hydrogen bonds. In contrast, in F127-DM, such physical cross-linking can occur only through water-mediated interactions.

Importantly, the terminal hydroxyl groups of native F127 can function as both hydrogen-bond donors and acceptors, whereas the ester oxygens of the terminal methacrylate groups in F127-DM act solely as hydrogen-bond acceptors.^50^ Consequently, methacrylation is expected to reduce the density of possible physical cross-links and increase the energetic barrier for gelation.

In addition, the substantially larger steric volume of the methacrylate group (∼100 Å^3^) compared to that of the hydroxyl group (∼19 Å^3^) is expected to decrease micellar packing efficiency, further affecting the gelation process.^20^ These molecular-level considerations are consistent with the SAXS and DSC results, which correlate directly with the rheological behavior and the morphological observations obtained by cryo-TEM.

Overall, gelation in Pluronic systems is primarily driven by the entropy gain associated with water release, but polymer conformational entropy and micelle interpenetration also play important roles. In native F127, hydrogen bonding between PEO chains and with surrounding water helps stabilize the interconnected micellar network. In contrast, methacrylated F127 lacks these specific interactions, likely requiring higher thermal energy to achieve micelle interpenetration and network formation. In addition, the flexible methacrylate end groups may enhance intramicellar mobility, favoring micelle stability over intermicellar interlocking, while steric effects from the methacrylate groups can further limit corona interpenetration. Together, these effects account for the increase in gelation temperature, the broader soft gel regime, an increased number of clusters with a shorter range of micellar packing and the formation of smaller, less ordered micellar clusters as the MD increases.

## Conclusions

This study systematically examined how methacrylation of Pluronic F127 alters its self-assembly, micellization, and gelation behavior prior to chemical cross-linking. By combining calorimetry, rheology, SAXS (including in situ heating), and cryo-TEM, we provide a comprehensive, multiscale picture of how terminal modification influences both thermodynamic and kinetic aspects of hydrogel formation. Methacrylation was found to have little effect on the thermodynamics of micellization, as reflected by unchanged micellization enthalpies and temperatures across the degrees of modification studied. This confirms that micellization remains primarily governed by PPO dehydration. In contrast, gelation is affected by methacrylation. Increasing the degree of methacrylation systematically shifts the gelation temperature to higher values and broadens the soft gel regime, indicating delayed and more gradual network formation. Structural characterization revealed that methacrylation weakens intermicellar interactions without altering the micelle core size or aggregation number. SAXS data show reduced micellar volume fraction, broader Bragg peaks, and smaller ordered domains with increasing methacrylation, consistent with hindered long-range ordering. Both equilibrium and in situ heating experiments demonstrate that methacrylation slows the development of ordered micellar lattices and promotes less coherent packing. Cryo-TEM directly visualizes this effect, revealing smaller and less ordered micellar clusters in methacrylated systems. Taken together, these results show that terminal methacrylation primarily impacts gelation through kinetic and structural mechanisms rather than micellization thermodynamics. The disruption of hydrogen bonding, reduced corona interpenetration, and increased end-group mobility collectively hinder micelle ordering and network formation. The gel diagrams presented here provide a practical framework for selecting methacrylation degree and operating conditions in applications where controlled gelation is critical, such as photo-cross-linkable hydrogels, additive manufacturing, drug delivery, and tissue engineering.

## Supplementary Material



## Notes

Amplitude stress sweep tests, a detailed description of the SAXS models and corresponding fitted curves, bar charts for micellization enthalpy, temperature at the peak of micellization and full width at half-maximum of the micellization peaks, temperature sweep rheological curves, additional rheological temperature steps experiment, a table of gelation temperatures, contour plots showing the temperature-dependent evolution of SAXS patterns, a table of calculated unit cell parameters, and gel permeation chromatograms of commercial F127 along with the molar fractions of methacrylated species, additional in situ heating curves, and cryo-TEM micrographs (PDF)
