# Supplementary material for: Self-Assembly and Gelation Behavior of Methacrylated PEO–PPO–PEO Triblock Copolymer Pluronic F127
Source: Langmuir. 2026 Mar 5;42(10):7452–62. doi: 10.1021/acs.langmuir.5c06879 (PMC13001103; doi:10.1021/acs.langmuir.5c06879)
Supplement: Supplementary file 1 [file la5c06879_si_001.pdf]

Supporting Information

Self-assembly and Gelation Behavior of Methacrylated PEO-PPO-PEO Triblock Copolymer Pluronic F127

Mateus P. Bomediano[a], Laura C. E. da Silva[b], Tomás S. Plivelic[c], Marcelo G. de Oliveira[a*]*

[a]Institute of Chemistry, University of Campinas, UNICAMP, 13083-970 Campinas, SP, Brazil.

[b]Brazilian Nanotechnology National Laboratory (LNNano), Brazilian Center for Research in Energy and Materials (CNPEM), Campinas, SP 13083-100, Brazil.

[c]MAX IV Laboratory, Lund University, P.O. Box 118, 221 00 Lund, Sweden.

*Authors to whom correspondence should be addressed (mgo@unicamp.br, tomas.plivelic@maxiv.lu.se)

Linear viscoelastic regime

Figures S1a and S1b show the amplitude stress and frequency sweeps, respectively, for 15 wt% F127, F127-DM50, and F127-DM100 at 35 °C. From the amplitude sweep (Figure S1a), the linear viscoelastic region (LVR) is identified, justifying the choice of 1 Pa as the applied stress for subsequent temperature sweep measurements. The frequency sweep (Figure S1b) demonstrates that the viscoelastic moduli remain stable over the range of oscillatory frequencies, supporting the selection of 1 Hz as the measurement frequency. These results confirm that the chosen parameters are within the LVR regime and appropriate for probing the gel behavior of the samples.

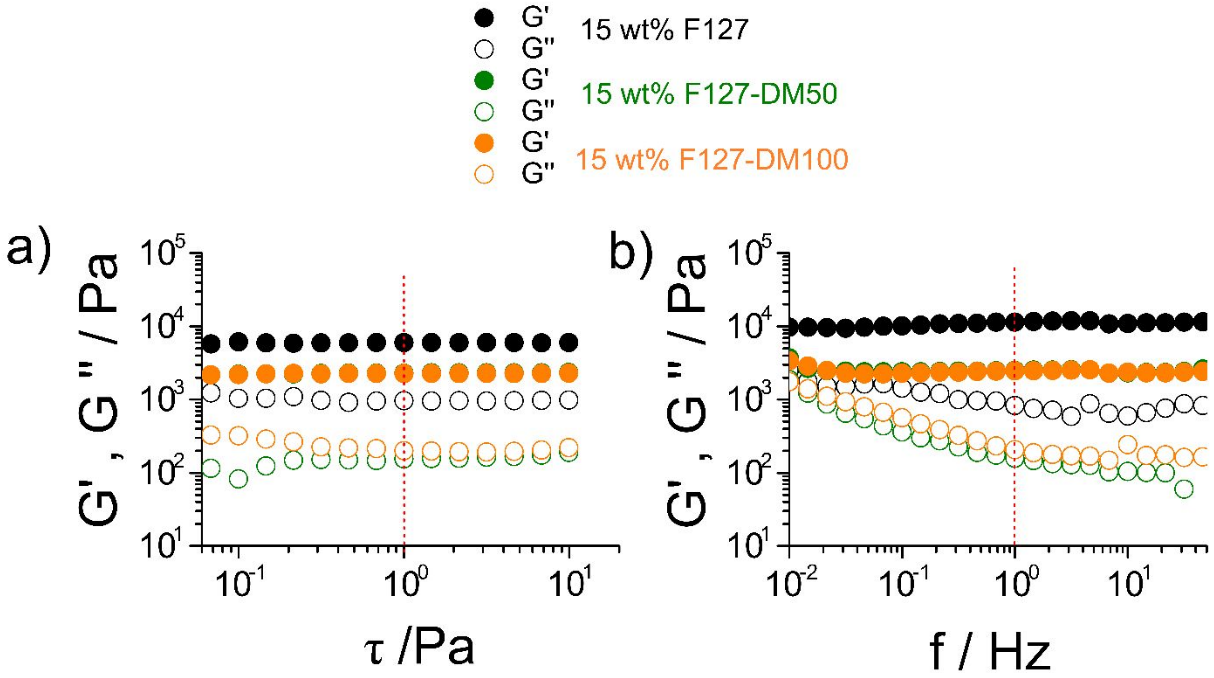

Figure S1. (a) Amplitude stress sweep of 15 wt% F127, F127-DM50, and F127-DM100 at 35 °C, showing the linear viscoelastic region (LVR) where the storage modulus (G′) and loss modulus (G″) are independent of applied stress. (b) Frequency sweep of 15 wt% F127, F127-DM50, and F127-DM100 at 35 °C, demonstrating the viscoelastic behavior over a range of oscillatory frequencies.

Micellization enthalpy, temperature at the peak of micellization and full width at half maximum of the micellization peaks

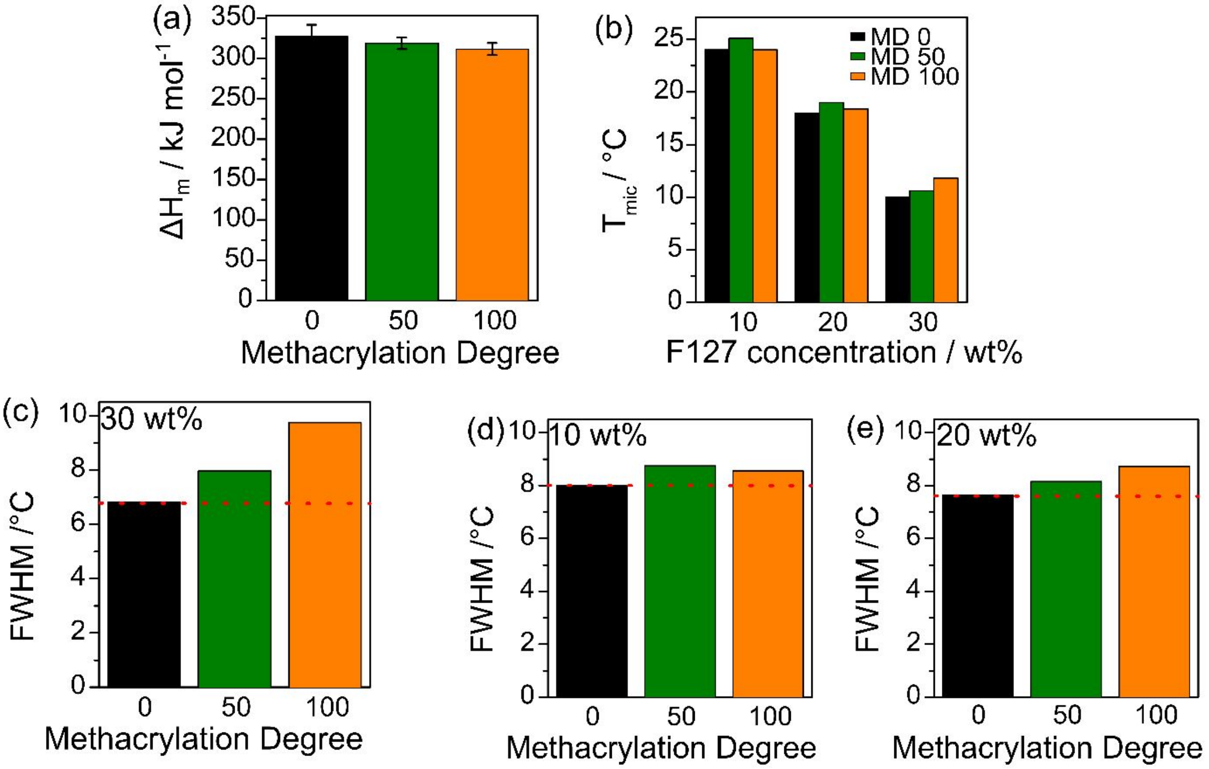

Figure S2. (a) Enthalpy of micellization (ΔH_m) and (b) peak micellization temperature (T_{mic}) as a function of polymer concentration and methacrylation degree. Full width at half maximum (FWHM) of the micellization peak at (c) 10 wt%, (d) 20 wt%, and (e) 30 wt%, illustrating the effect of methacrylation degree on the sharpness of the micellization transition.

SAXS Fitting and Simulation for block-copolymer

For micelles in water solution (10 wt% Pluronic), the scattering data were fitted using a block copolymer micelle form factor[1] combined with a hard-sphere Percus-Yevick structure factor, as described in Eq. (1). This model assumes micelles composed of a homogeneous hydrophobic spherical core surrounded by a corona of Gaussian polymer chains, with intermicellar interactions accounted for by the structure factor.

$$I(q) = \Bigg(N_{agg}^2 \beta_{PPO}^2 \left[\frac{3(\sin(qR_c) - qR_c \cos(qR_c))}{(q R_c)^3} \right]^2 + N_{agg} \beta_{PEO}^2 \frac{2\left(e^{-q^2 R_g^2} + q^2 R_g^2 - 1\right)}{\left(q^2 R_g^2\right)^2} + 2N_{agg}^2 \beta_{PPO} \beta_{PEO} \left[\frac{3(\sin(qR_c) - qR_c \cos(qR_c))}{(qR_c)^3} \right] \left[\frac{1 - e^{-q^2 R_g^2}}{q^2 R_g^2} \right] \left[\frac{\sin\left(q(R_c + dR_g)\right)}{q(R_c + dR_g)} \right] + N_{agg}(N_{agg} - 1) \beta_{PEO}^2 \left[\frac{1 - e^{-q^2 R_g^2}}{q^2 R_g^2} \right]^2 \left[\frac{\sin\left(q(R_c + dR_g)\right)}{q(R_c + dR_g)} \right]^2 \Bigg) \frac{1}{1 + 24\Phi G(2qR_{eff})/2qR_{eff}} \quad (1)$$

where

$$\beta_{PPO} = \frac{V_{PPO}}{2}(\rho_{PPO} - \rho_{water}) \quad (2)$$

$$\beta_{PEO} = V_{PEO}(\rho_{PEO} - \rho_{water}) \quad (3)$$

Here, ρ is scattering length density of each component. The PPO block volume is $V_{PPO}/2$ (assuming a diblock model). Each PEO chain is represented as an individual unit, with 100 ethylene oxide units and volume V_{PEO}. The aggregation number N_{agg} is the number of diblock chains per micelle. This value was divided by two for triblock copolymers. $R_c$is the micellar core radius. $R_g$is the radius of gyration of the PEO chains. $R_{eff}$is the hard-sphere radius, and Φ is the volume fraction. $G(A)$ is defined by, where $A = 2qR_{eff}$:

$$G(A) = \frac{\alpha(\sin A - A\cos A)}{A^2} + \frac{\beta\,[2A\ \sin A + (2 - A^2)\ \cos A - 2\,]}{A^3} + \frac{\gamma\left[-A^4 \cos A + 4\{(3A^2-6)\cos A + (A^3-6A)\sin A + 6\}\right]}{A^5}$$

$$\text{where } \alpha = \frac{(1+2\Phi)^2}{(1-\Phi)^4}\,;\beta = -6\Phi\frac{(1+\Phi/2)^2}{(1-\Phi)^4};\gamma = \frac{\Phi\alpha}{2}$$

The SAXS data and corresponding fittings for 10 wt% F127, F127-DM50, and F127-DM100 at 50 °C are shown in Figure S3. The red lines represent the best fits obtained using the form factor/structure factor model described above. The fitted parameters (core radius (R_c), aggregation number (N_{agg}), and volume fraction (Φ) are summarized in Table 3 of the main text.

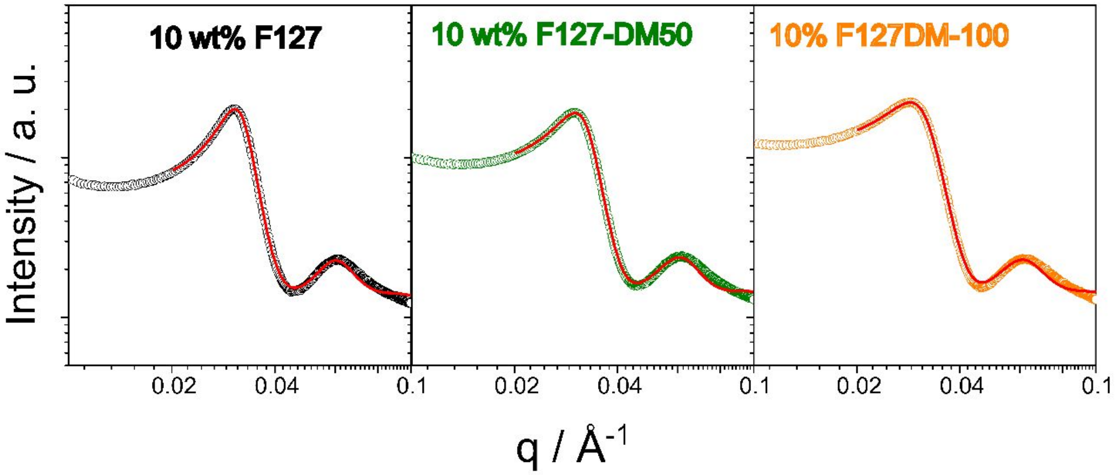

Figure S3. SAXS curves at for 10 wt% F127, F127-DM50 and F127-DM100 samples at 50 ˚C. The red lines represent the fitted curves using form factor for block copolymer micelles combined with a structure factor based on the hard-sphere Percus-Yevick approximation.

Rheological measurements

All temperature-dependent representative measurements of G' and G'' for the studied hydrogel concentrations are presented in Figure S4. The gelation temperature (T_{gel}) was determined from the crossover of the moduli (G′ = G″) and is discussed in the main text.

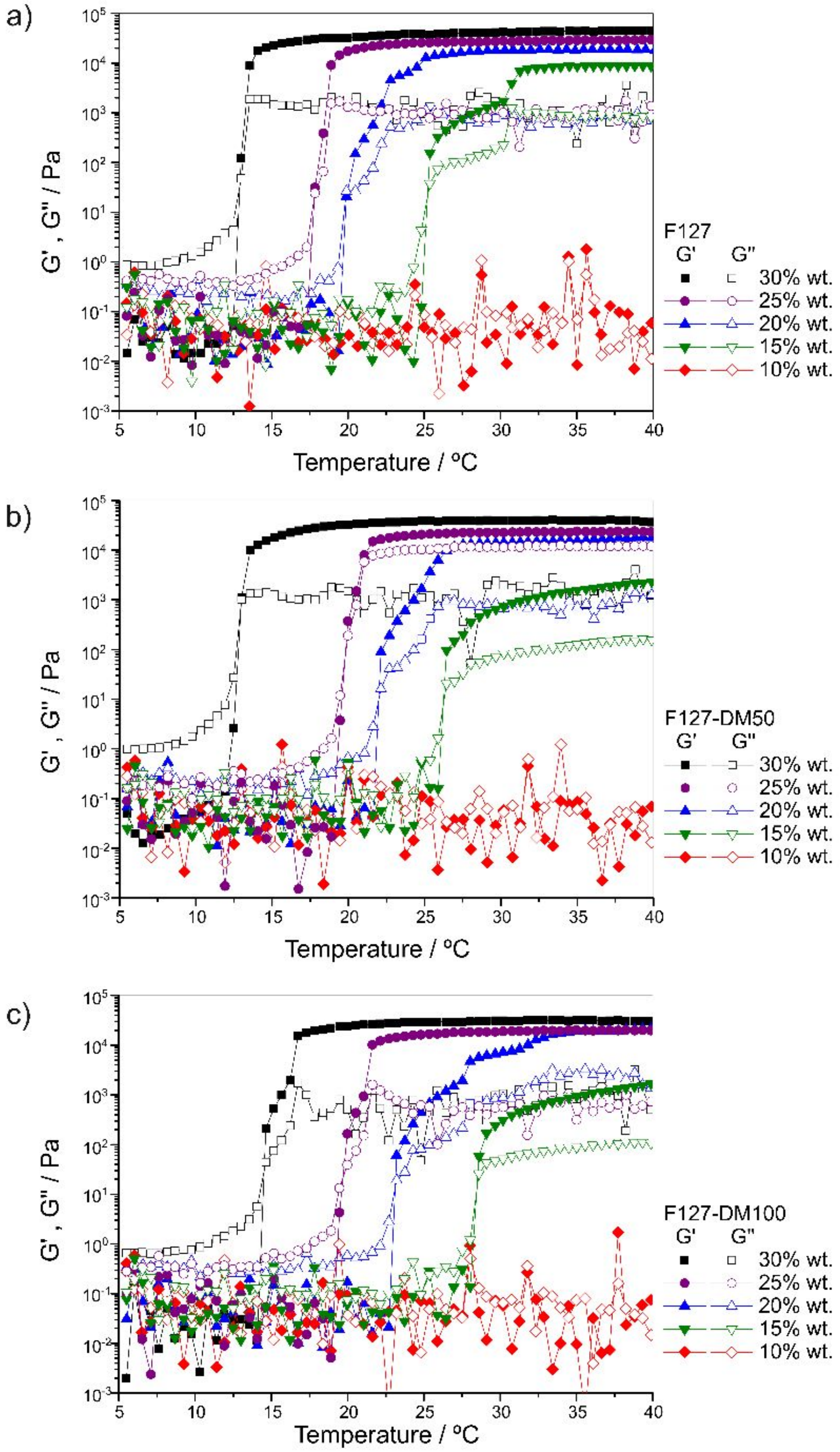

Figure S4. Temperature sweeps showing the storage modulus (G') and loss modulus (G") as a function of temperature for F127, F127-DM50, and F127-DM100 at concentrations of 10 wt%, 15 wt%, 20 wt%, 25 wt% and 30 wt%.

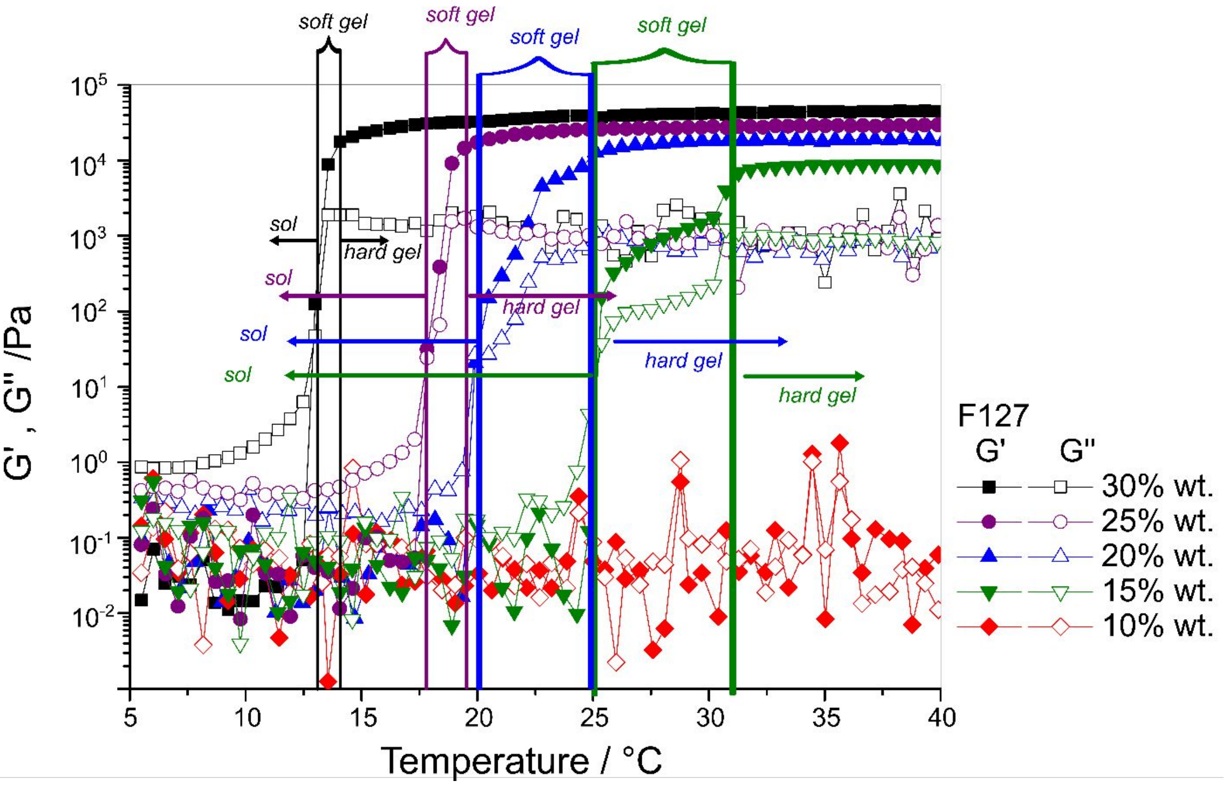

Figure S5. Temperature sweeps showing the storage modulus (G') and loss modulus (G'') as a function of temperature for F127 at concentrations of 10 wt%, 15 wt%, 20 wt%, 25 wt% and 30 wt%. Three distinct regions are indicated: a sun region, where the loss modulus exceeds the storage modulus (G' > G''), a region we are calling soft gel, where G' > G'', which begins after the G' and G'' curves cross (G' = G''), and ends when the G' and G'' moduli reach plateau values, and a hard gel region, which comes into existence after the plateaus are established.

Temperature steps experiment

To address concerns regarding potential bias from the heating rate, temperature step experiments were performed on 15 wt% F127. As seen in Figure S6, the sample was heated until the soft gel crossover (G′ = G″) was reached, and the temperature was then held constant for 20 min. During this isothermal hold, the moduli continued increasing albeit at a lower rate (G′ ≈

10^2 - 10^3 Pa). Heating was subsequently resumed at 1 °C min^{-1}, leading to a further increase in moduli until a final plateau at $\approx 10^4$ Pa, consistent with the hard gel regime measured previously.

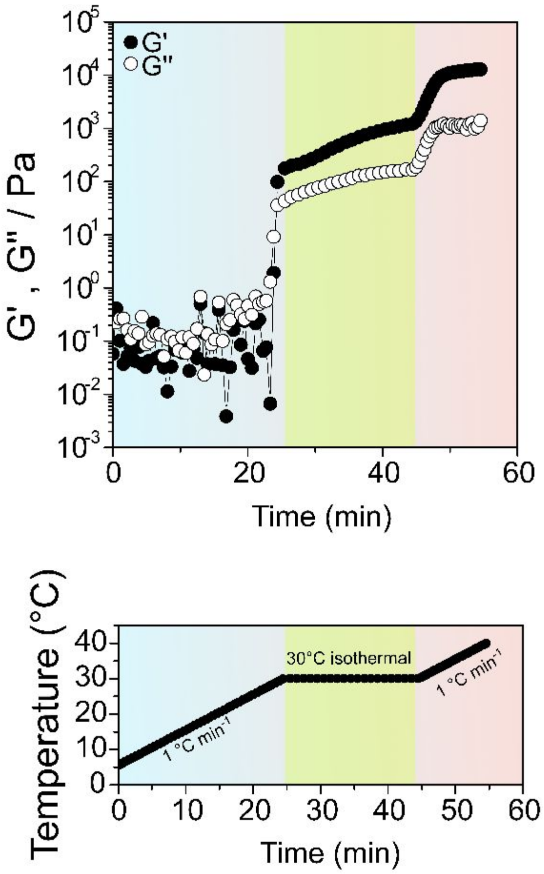

Figure S6. Rheological temperature sweep of 15 wt% F127, performed at a heating rate of 1 ˚C min^{-1} up to the crossover point of G' and G" (30 ˚C, blue region). Time sweep measurements were subsequently conducted at 30 ˚C for 20 min (yellow region). A final heating step was then applied at 1 ˚C min^{-1} up to 40 ˚C (pink region). The bottom panel shows the temperature profile applied over time, including the initial heating ramp, the isothermal plateau at 30 ˚C, and the final heating ramp.

Gelation Temperature

The critical gelation temperature *(cgt)* determined by DSC and the gelation temperature (T_{gel}), obtained from rheological measurements are summarized in Table S1, discussion is provided in the main text.

Table S1. Critical gelation temperature (*cgt*) and T_{gel} for 30 wt% F127, F127-DM50, and F127-DM100.

Sample	*cgt* (˚C)	T_{gel} (˚C)
F127	*12.9*	*13.0*
F127-DM50	*14.3*	*13.5*
F127-DM100	*15.5*	*15.1*

Contour plots of temperature-dependent evolution of SAXS

Figure S7 shows the contour plots of temperature-resolved SAXS data for 20 and 30 wt% F127, F127-DM50, and F127-DM100 samples recorded from 10 to 50 °C. The plots show the evolution of the Bragg peaks associated with the formation of ordered micellar phases upon heating.

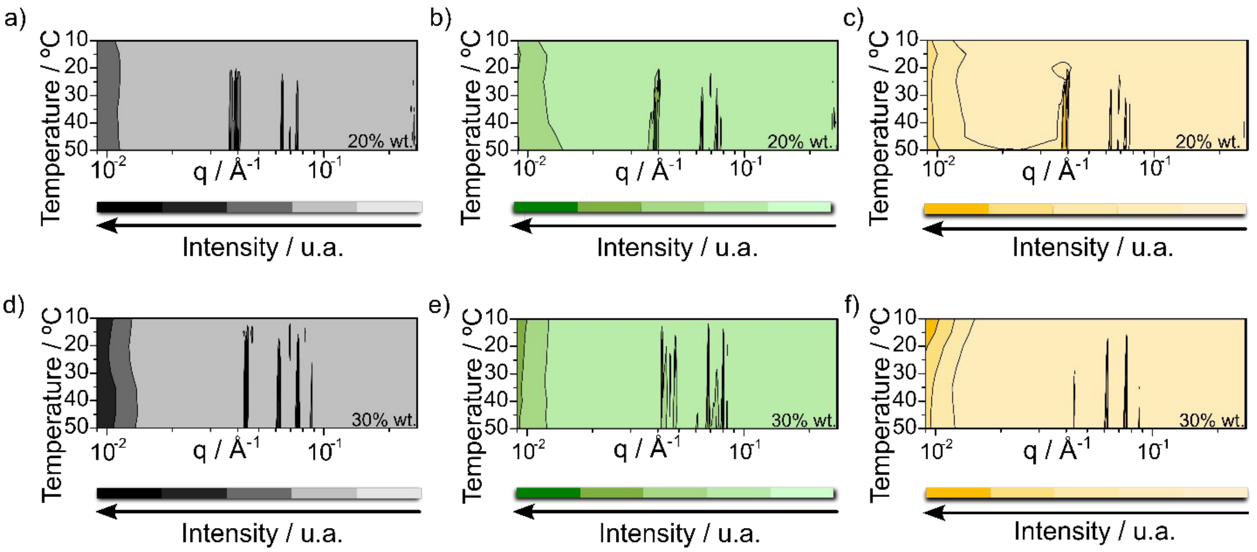

Figure S7. Contour plots of temperature-dependent evolution of SAXS profile of 20 wt% a) F127, b) F127DM50, and c) F127DM100 and 30 wt% d) F127, e) F127DM50, and f) F127DM100.

The peak positions used for the analysis were obtained directly from the SAXS curves, as visualized in the contour plots. The corresponding d-spacing values were calculated using $d = 2\pi/q$. All the cell parameters shown in Table S2 were calculated from the curves of $d_{hkl}\ vs.\ \frac{1}{\sqrt{h^2+k^2+l^2}}$ at a specific temperature, the cell parameter (a) is the slope of a well-the linear curve fitting.

Table S2. Cell parameter (a) and aggregation number (N_{agg}) of 20 wt% and 30 wt% F127, F127-DM50 and F127-DM100 samples

Sample	Phase	Concentration (wt%)	Temperature (˚C)	a (nm)	N_{agg}
**F127**	BCC	20	25	21.96 ± 0.04	61 ± 1
			50	22.20 ± 0.10	65 ± 3
		30	25	20.21 ± 0.03	84 ± 1
			50	20.40 ± 0.10	87 ± 4
	FCC	20	25	27.60 ± 0.10	63 ± 3
			50	27.70 ± 0.10	63 ± 3
		30	25	25.65 ± 0.08	88 ± 1
			50	No clear bcc	—
**F127-DM50**	BCC	20	25	22.11 ± 0.05	63 ± 1
			50	No clear bcc	—
		30	25	20.34 ± 0.08	85 ± 2
			50	20.60 ± 0.10	89 ± 5
	FCC	20	25	28.00 ± 0.10	66 ± 3
			50	28.10 ± 0.20	67 ± 4

		30	25	26.10 ± 0.10	92 ± 1
			50	26.00 ± 0.20	90 ± 4
**F127-DM100**	BCC	20	25	22.22 ± 0.05	66 ± 2
			50	No clear bcc	—
		30	25	20.31 ± 0.06	86 ± 2
			50	20.48 ± 0.05	88 ± 2

F127 gel permeation chromatography (GPC) and molar fraction of methacrylated species

Figure S8a shows the GPC chromatogram of F127, with detector response as a function of retention volume. A bimodal curve is observed, corresponding to the triblock copolymer (lower retention volume) and a minor diblock fraction (higher retention volume). The area under each peak, shown in Figure S8b, indicates the F127 composition: 90.3% triblock and 9.7% diblock.

After the methacrylation procedure, each Pluronic F127 molecule can have 0, 1, or 2 methacrylate groups. Assuming that the two terminal hydroxyls react independently[2], the fraction of molecules with n = 0, 1, or 2 methacrylates can be estimated as:

$$x_0 = (100 - MD)^2\ ,\ x_1 = 2MD(100 - MD),\ x_2 = (MD)^2$$

where MD is the methacrylation degree (%) determined by ^{1}H-NMR. In this work, MD values of 54 ± 3% and 95 ± 2% were obtained. Fractions were corrected for the diblock content by multiplying by the respective triblock and diblock fractions. Figure S8c–d shows the molar fractions of F127 molecules carrying 0, 1, or 2 methacrylate groups (i.e., terminal hydroxyl groups that have been methacrylated) for F127-DM50 and F127-DM100

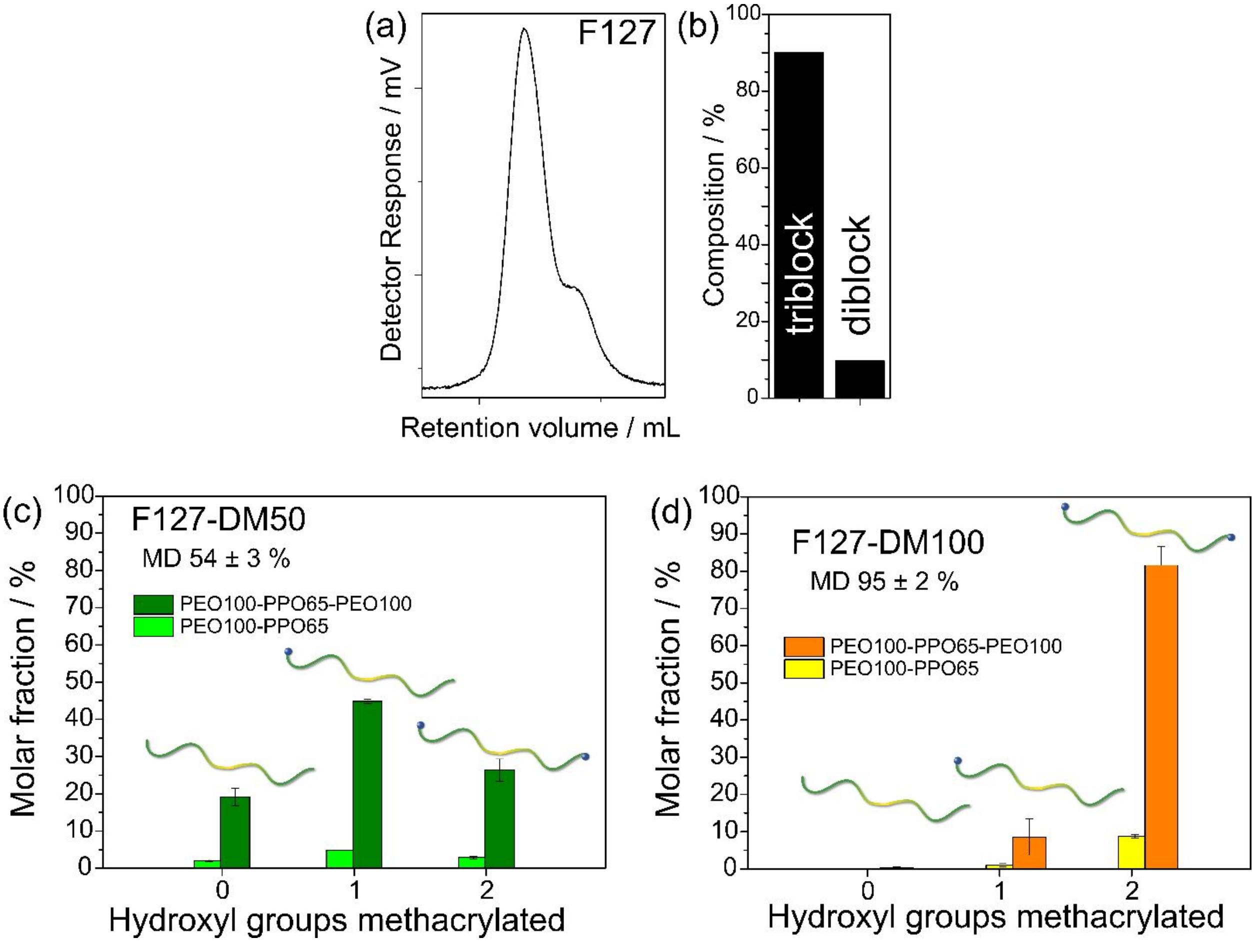

Figure S8. (a) GPC chromatogram of F127 showing the detector response as a function of retention volume. (b) Composition of F127 determined from the area under each peak of the GPC chromatogram. (c–d) Molar fractions of F127 molecules carrying 0, 1, or 2 methacrylate groups for F127-DM50 (c) and F127-DM100 (d).

In situ heating SAXS

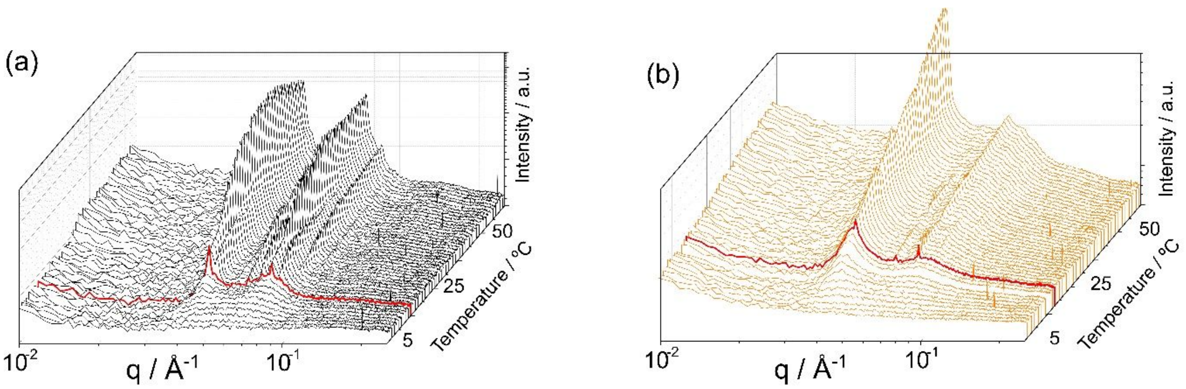

Figure S9. Three-dimensional representation of the temperature-dependent evolution of SAXS profiles during *in situ* heating of 30 wt% (a) F127 and (b) F127-DM100.

Cryo-TEM

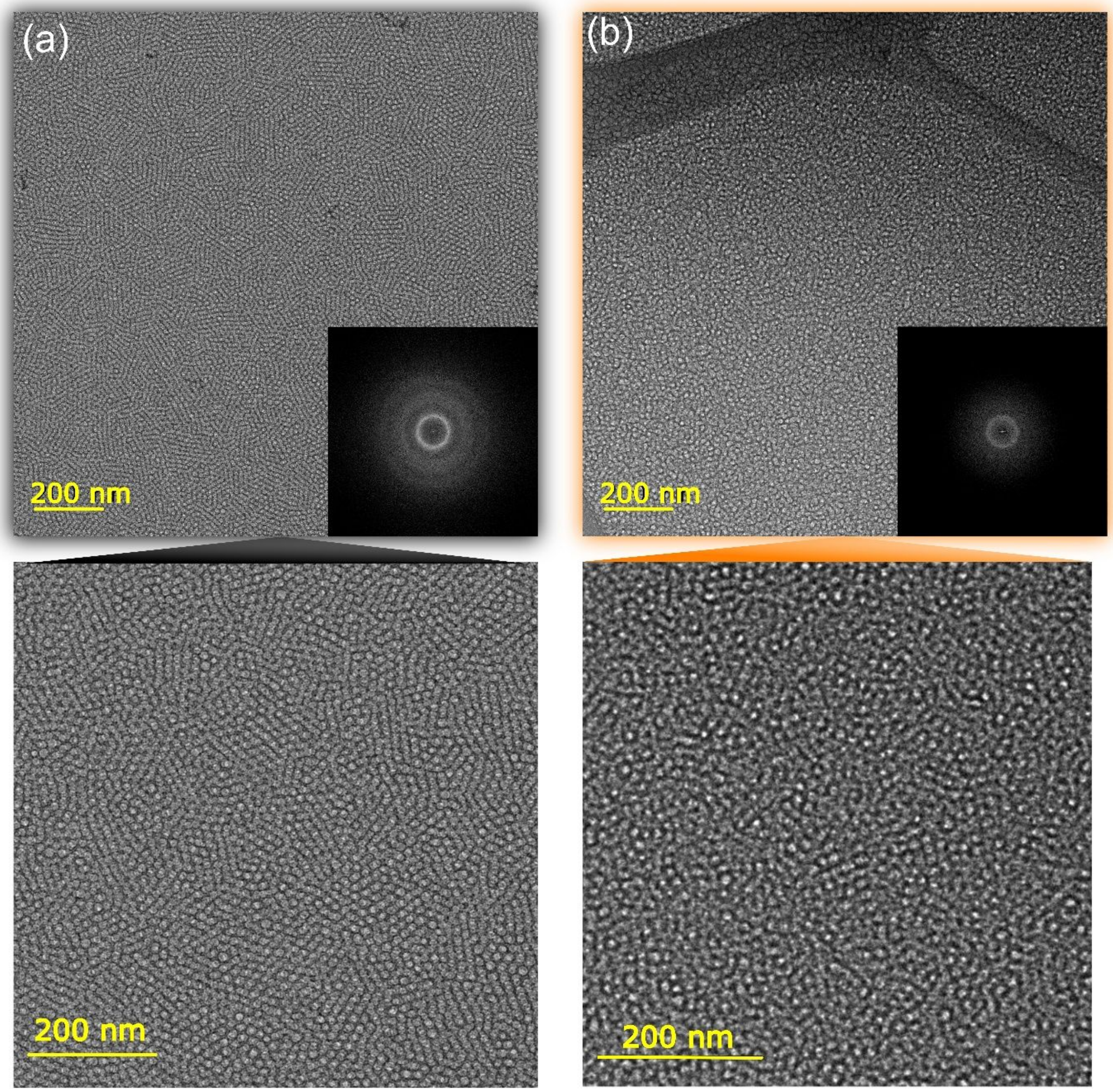

Figure S10. Cryo-TEM micrographs and corresponding magnified views of 20 wt% solutions of (a) F127 and (b) F127-DM100. The corresponding fast Fourier transforms (FFTs) are shown in the lower right corner of each image.

Cryo-TEM 30 wt% F127, F127-DM50, and F127-DM100

Figure S11 presents cryo-TEM micrographs of 30 wt% F127, F127-DM50, and F127-DM100 samples. It is important to emphasize that these micrographs were prepared using a different specimen preparation protocol than that described in the Main Text (Figure 5).The 30 wt% formulations are highly viscous, even at 4 °C, which makes conventional cryo-TEM grid preparation challenging. To reduce viscosity and enable proper thinning, the samples were loaded into a plastic syringe and equilibrated in a water bath at a temperature above the critical gelation temperature (cgt, ~50 °C) for 30 min.

For grid preparation, a TEM grid was held with tweezers and positioned between two wet filter papers to create a saturated humid atmosphere. This assembly was placed on a heating plate maintained at 50 °C. After 30 min of thermal equilibration, approximately 50 μL of the pre-heated sample was deposited onto the grid using the warmed syringe. A razor blade was then used to gently drag the sample across the grid surface and toward the filter paper to achieve thinning. Following deposition, the specimen was maintained in the humid chamber for 3 min to allow structural relaxation after shear. The grid was then manually plunge-frozen in liquid ethane. This preparation method has been successfully applied to other highly viscous systems[3] and was employed here to enable improved visualization of the hard gel samples.

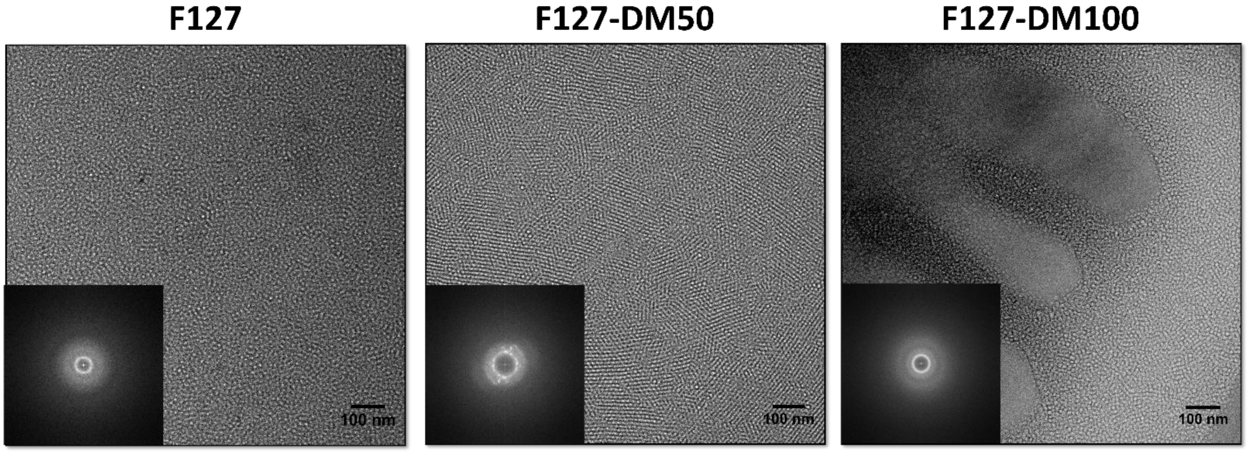

Figure S11. Cryo-TEM micrographs of 30 wt% solutions of F127, F127-DM50 and F127-DM100.

References

(1) Pedersen, J. S.; Gerstenberg, M. C. *Communications to the Editor Scattering Form Factor of Block Copolymer Micelles*; UTC, 1996; Vol. 29. https://pubs.acs.org/sharingguidelines.

(2) Perin, G. B.; Felisberti, M. I. A Simple Mathematical Model for the Structural Description of Polyesters Based on Glycerol. *Macromolecules* **2025**, *58* (16), 8581–8590. https://doi.org/10.1021/acs.macromol.5c01193.

(3) Nogueira, H.C.N.; da Silva, L.C.E.; Plivelic, T.S.; Lutz-Bueno, V; and Sabadini, E. Alkane selection as a critical factor in structuring reverse wormlike micelles. *Journal of Colloid and Interface Science* **2025**, *684*, 170-180. https://doi.org/10.1016/j.jcis.2025.01.085
